# Serum Levels of Netrin-4 and Its Association With Hepatocellular Carcinoma: Results From a Case-Control Study

**DOI:** 10.7759/cureus.43844

**Published:** 2023-08-21

**Authors:** Vennela Jyothi, Biju Pottakkat, Balasubramaniyan V, Surendra Kumar Verma

**Affiliations:** 1 Surgical Gastroenterology, Jawaharlal Institute of Postgraduate Medical Education and Research, Puducherry, IND; 2 Biochemistry, Jawaharlal Institute of Postgraduate Medical Education and Research, Puducherry, IND; 3 Pathology, Jawaharlal Institute of Postgraduate Medical Education and Research, Puducherry, IND

**Keywords:** alpha-fetoprotein, angiogenesis, hcc, biomarker, netrin4

## Abstract

Background: Angiogenesis plays a vital role in the progression of hepatocellular carcinoma (HCC) by contributing to tumor growth and metastasis. Netrin-4 (NTN4) is a secreted glycoprotein that regulates angiogenesis and maintains endothelial homeostasis. There were no studies found focusing on the value of NTN4 as a serum biomarker for the diagnosis and prognosis of HCC. In this study, we aimed to investigate the systemic expression of NTN4 in patients with HCC. We also explore the association of NTN4 with major clinicopathological and biochemical characteristics of HCC.

Methods: A total of 116 patients with HCC and 44 healthy volunteers were recruited in this case-control study. Clinical characteristics and liver function parameters were recorded among the study subjects. The levels of α-fetoprotein (AFP) were quantified in patients with HCC. The serum levels of NTN4 (pg/ml) were estimated by enzyme-linked immunosorbent assay (ELISA).

Results: The median NTN4 levels were significantly decreased in patients with HCC when compared to control subjects (p < 0.0001). There was no difference between NTN4 levels in AFP-positive patients and AFP-negative patients (p = 0.39). Of note, NTN4 levels were significantly decreased in HCC patients with metastasis (p < 0.02) and portal vein invasion (p < 0.04). Further, NTN4 levels were significantly reduced in HCC patients with Child-Pugh C score (p < 0.05). The receiver operating characteristic curve for serum levels of NTN4 in the HCC group and control group was generated. At a cut-off of 30 pg/ml, the sensitivity and specificity for NTN4 were 80% and 82%, respectively, with an AUC of 0.894.

Conclusions: Low levels of NTN4 were associated with increased tumor aggressiveness and metastasis in HCC. Estimation of circulating NTN4 has prognostic value as a minimally invasive biomarker in HCC. Future studies might shed the role of NTN4 in the development of HCC.

## Introduction

Hepatocellular carcinoma (HCC) is one of the most aggressive malignancies in humans and significantly contributes to cancer-related mortality [1]. Several etiological factors, including chronic hepatitis B virus (HBV) and hepatitis C virus (HCV) infections, alcohol-related liver disease, cirrhotic disease, smoking, nonalcoholic fatty liver, obesity, diabetes, and exposure to carcinogenic toxins, have been recognized to increase the risk of HCC development [1,2]. Globally, close to a million individuals were diagnosed with HCC in 2020 alone, a number which is predicted to increase by 55% in 2040 [3]. Approximately 0.83 million worldwide deaths are attributed to HCC every year. While HBV and HCV are the most prevalent risk factors for HCC development in Asian countries, chronic inflammation and injury to the liver through nonalcoholic fatty liver disease (NAFLD) are the prime causes of the disease in Western and European countries [4]. Although surgical resection of the tumor is a major treatment option, over 40-70% of patients exhibit an intrahepatic recurrence of cancer after surgery [5]. Despite serving as an effective treatment modality in cirrhotic patients who developed HCC, the prospect of liver transplantation is limited due to organ shortage [6]. It should be noted that, unlike many other gastrointestinal malignancies, more than 50% of HCC patients are asymptomatic at presentation, and often exhibit a delay in the manifestation of the advanced stages of the disease, resulting in a dismal prognosis [7]. Considering that the five-year survival rate is as low as 10-20% in HCC patients [8], it is imperative to identify the biomarkers associated with tumor formation, and metastasis for the early detection and effective treatment of HCC. Alpha-fetoprotein (AFP) is the most widely used clinical marker for HCC and has served as a robust predictor of HCC progression. However, AFP-dependent prognosis is severely hampered in HCC patients who exhibit negative AFP results, further highlighting the need for the identification of novel circulating biomarkers that are altered in both AFP-positive and negative patients [9]. HCC is a highly vascularized tumor, thereby making angiogenesis play a vital role in tumor growth and metastasis. Multiple proteins participate in the tumor-induced angiogenesis in HCC, which has shifted the focus toward the investigation of such molecules as potential prognostic biomarkers [10]. Netrin-4 (NTN4) is a member of the family of laminin-related secreted glycoproteins, which dynamically regulate angiogenesis based on the tumor microenvironment [11]. NTN4 shares a structural similarity to the short arms of the laminin β chains and hence is also referred to as β-netrin [12]. Physiologically, NTN4 is largely expressed in endothelial cells, where it has been reported to maintain endothelial homeostasis and vascular health [13]. Notably, alteration in NTN4 expression has been implicated in a range of human malignancies and has been proposed as a prognostic marker in some cancers, where it was reported to inhibit angiogenesis and delay tumor development. While a batch of evidence indicates the decreased expression of NTN4 in breast, pancreas, prostate, cervical, and colon cancer, elevated levels of this protein have also been documented in gastric cancer [12]. Moreover, the majority of the data on NTN4 expression in various cancers have been demonstrated in experimental models [14-16], and its role and clinical significance in human malignancies remain uncertain. In particular, the prognostic value of NTN4 as a biomarker in early detection and its association with HCC pathogenesis has not been explored. In this study, we aimed to investigate the expression of NTN4 in patients with HCC and determine its correlation with the clinical features and biochemical parameters, to understand if it has prognostic value in HCC.

## Materials and methods

Study design

This study aimed to investigate the relationship between serum NTN4 levels and HCC with the hypothesis that in patients with HCC, the levels of NTN4 will be below normal. This case-control study was conducted in the Departments of Surgical Gastroenterology, Biochemistry, and Pathology from October 2017 to December 2020 at Jawaharlal Institute of Postgraduate Medical Education and Research. The study was conducted after obtaining approval from the institute's ethics committee for human studies based on the ethical guidelines of the Helsinki Declaration. The purpose of the study was explained to the eligible participants, and written informed consent was obtained. The cohort subjects were patients with HCC (n = 116) aged between 18 and 80 years admitted to the Department of Surgical Gastroenterology. Healthy participants with normal physical and mental health, normal liver function tests, and no recent illness were recruited as controls (n = 44).

Diagnosis of HCC and assessment of clinical characteristics

HCC was diagnosed by the standard criteria of the European Association for the Study of the Liver (EASL). Ultrasonography (USG), magnetic resonance imaging (MRI), computed tomography (CT), α-fetoprotein (AFP) levels, histology, etc., were used for the diagnosis of HCC. Tumor characteristics on radiology (portal vein invasion, vascular infiltration, metastasis, number of lesions, and tumor size) were evaluated. The severity of liver disease among HCC patients was assessed by the Child-Pugh score, and the tumors were staged according to the Barcelona Clinic Liver Cancer (BCLC) staging system. Patients with an uncertain diagnosis of HCC, rare variants of HCC, patients with inadequate contrast imaging, and severely ill patients were excluded from the study. Socio-demographic characteristics, clinical characteristics, behavioral characteristics, comorbidity, treatment history, and outcomes were recorded.

Sample collection and assessment of biochemical parameters

A venous blood sample of 5 ml was drawn from each study participant in a precooled tube. Collected blood samples were centrifuged (REMI-R8M, REMI, Mumbai, India) at 3500 rpm for 10 minutes, and serum was separated. Routine biochemistry tests were performed immediately. Biochemical parameters such as aspartate transaminase (AST), alkaline phosphatase (ALP), gamma-glutamyl transferase (GGT), albumin, total protein, and bilirubin concentrations were analyzed in the serum by AU680 Beckman Coulter autoanalyzer (Brea, CA). Serum AFP level was measured by chemiluminescence using the ADVIA Centaur CP immunoassay system (Siemens Healthineers, Erlangen, Germany) at the time of diagnosis. The remaining samples (serum) were stored at −80°C for quantification of NTN4.

Enzyme-linked immunosorbent assay

The serum levels of NTN4 were measured using the enzyme-linked immunosorbent assay (ELISA) method with the commercially available kit (Genxbio Health Sciences Pvt. Ltd., Delhi, India) as per manufactures instructions. Samples or standards were added to the ELISA wells, along with 50 µL of streptavidin-horseradish peroxidase and 10 µL of antibody. The ELISA plate was incubated at 37ºC for 60 minutes. After washing five times with wash buffer, chromogen reagents A and B were added to the well and incubated for 10 minutes at 10ºC. The color reaction was stopped using a stop solution, after which the optical density was estimated at 450 nm using a SpectraMax Microplate reader (Molecular Devices, San Jose, CA). The NTN4 levels were measured as pg/ml.

Statistical analysis

Statistical analysis was performed using Statistical Package for the Social Sciences (SPSS version 20, IBM Corp., Armonk, NY) and GraphPad Prism 6.0 (GraphPad Software, San Diego, CA). Qualitative variables were presented as numbers and percentages while quantitative variables were presented as mean ± SD and median (interquartile range (IQR)) for Gaussian and non-Gaussian distribution, respectively. For the comparison of the two groups, a two-tailed unpaired t-test or a Mann-Whitney U test was used wherever appropriate. Spearman's correlation was performed to assess the correlation between the study parameters. The receiver operating characteristic (ROC) curve was used to assess the best cut-off value of NTN4 between the two groups with its sensitivity, specificity, and area under the curve (AUC). A p-value < 0.05 was considered statistically significant.

## Results

Clinical characteristics of the study subjects

A total of 129 patients with HCC were considered for the study. Thirteen patients who did not meet the inclusion criteria because of the poor quality image were excluded and hence 116 patients were included in the analysis. Among 116 patients with HCC, there were 98 (84%) males and 18 (16%) females with a median age of 60 (50-66) years. These patients were compared with normal healthy controls (n = 44). Among controls, there were 24 (54%) males and 20 (46%) females with a median age of 33 (27-36) years. A higher proportion of males were found in patients with HCC compared to females, which was statistically significant (p < 0.001). The clinical characteristics of patients with HCC recruited in this study are summarized in Table 1. Among the patients with HCC, 53.4% of the population had a single lesion, 15.5% had two lesions, 5.2% had three lesions, and 25.9% had multifocal lesions. Of the population, 42.2% had a maximum tumor size of ≤5 cm.

**Table 1:** Clinical characteristics of patients with HCC (N = 116) HCC: hepatocellular carcinoma; BCLC: Barcelona Clinic Liver Cancer; HBV: hepatitis B virus; HCV: hepatitis C virus; * due to cirrhosis and tumor infiltration.

Variables	Category	HCC, n (%)
BCLC staging	A	15 (12.9%)
	B	30 (25.9%)
	C	60 (51.7%)
	D	11 (9.5%)
Child-Pugh class	A	64 (55.2%)
	B	42 (36.2%)
	C	10 (8.6%)
Hepatitis	HBV positive	24 (20.7%)
	HCV positive	13 (11.2%)
Cirrhosis	Present	40 (34.5%)
Portal hypertension*	Present	56 (48.3%)
Ascites	Present	39 (33.6%)
Encephalopathy	Present	7 (6%)
Esophageal varices	Present	48 (41.4%)

Biochemical parameters in patients with HCC and healthy controls

The serum concentrations of total and direct bilirubin, AST, gamma-glutamyl transferase (γGT), total protein (p < 0.001), and ALP (p < 0.001) were significantly elevated in patients with HCC compared to healthy controls (Table 2). Serum albumin concentrations were not significantly decreased in patients with HCC compared to healthy controls (p = 0.09) (Table 2).

**Table 2:** Biochemical parameters in patients with HCC and healthy controls (N = 160) HCC: hepatocellular carcinoma; AST: aspartate transaminase; γGT: gamma-glutamyl transferase; ALP: alkaline phosphatase; IQR: interquartile range; p-value < 0.05 is statistically significant.

Parameters	HCC (n = 116)	Healthy controls (n = 44)	P-value
	Median (IQR)	Median (IQR)	
Total bilirubin (mg/dL)	1.2 (0.7-2.1)	0.5 (0.3-0.6)	<0.000
Direct bilirubin (mg/dL)	0.4 (0.2-0.8)	0.2 (0.1-0.3)	<0.000
Total protein (mg/dL)	6.9 (6.1-7.6)	5.7 (4.8-6.5)	<0.000
Albumin (gm/dL)	3.2 (2.7-3.6)	3.3 (2.5-3.5)	0.09
AST (IU/L)	73.5 (47.0-145.2)	24.0 (20.0-28.0)	<0.000
γGT (IU/L)	85.5 (57.0-185.2)	22.0 (17.0-27.7)	<0.000
ALP (IU/L)	154.5 (106.0-254.5)	121.5 (91.2-158.7)	<0.001

Levels of NTN4 in patients with HCC and healthy controls

**Figure 1:**
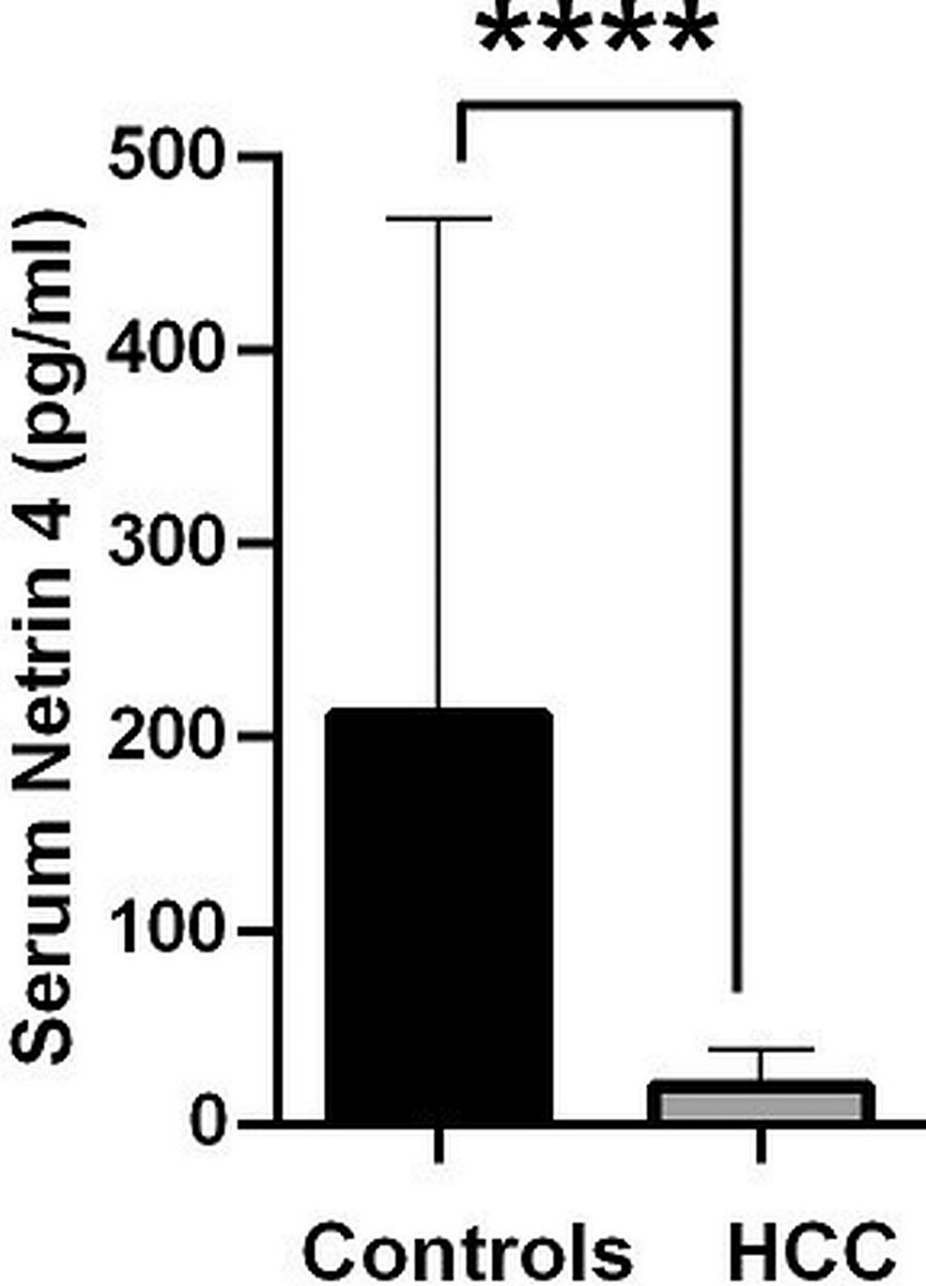
Serum NTN4 levels in patients with HCC (n = 110) and healthy controls (n = 39) Values are expressed as controls - mean = 211.9 and SD = 256.3; HCC - mean = 19.64 and SD = 19.41; p < 0.0001 compared to controls. NTN4: netrin-4; HCC: hepatocellular carcinoma.

Six patients in the HCC group and five patients in the control group were excluded, as their NTN4 values were not properly recorded. The levels of NTN4 in patients with HCC (n = 110) were found to be markedly reduced compared to controls (n = 39) (Figure 1 and Table 3).

**Table 3:** NTN4 levels in patients with HCC and healthy controls NTN4: netrin-4; HCC: hepatocellular carcinoma; IQR: interquartile range; p-value < 0.05 is statistically significant.

Variable	Study group	Mean ± SD	Median (IQR)	P-value
NTN4 pg/ml	HCC (n = 110)	19.64 ± 19.41	14.7 (6.8-25.8)	<0.0001
	Controls (n = 39)	211.9 ± 256.3	119.8 (30.8-216.8)	

Serum levels of NTN4 according to Child-Pugh class in patients with HCC (n = 110)

**Figure 2:**
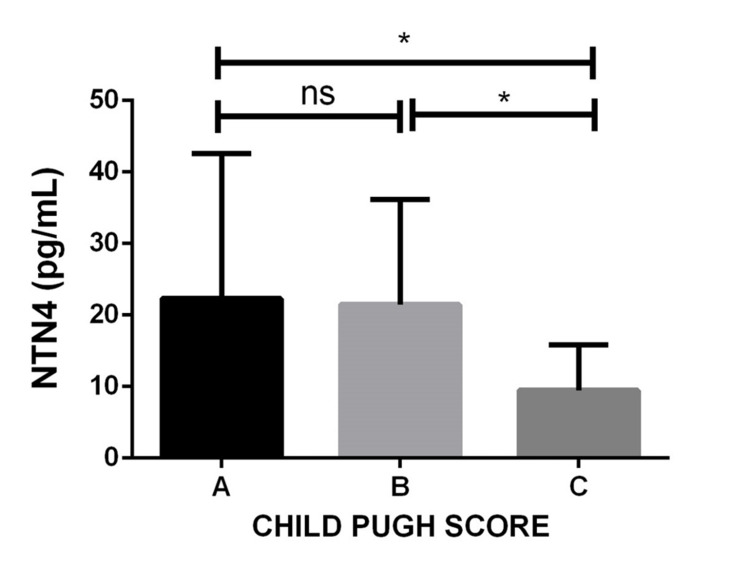
Serum NTN4 levels according to the Child-Pugh class in patients with HCC Values are expressed as mean ± standard deviation, * p < 0.05 Child-Pugh class B vs. C; Child-Pugh class A vs. C. NTN4: netrin-4; HCC: hepatocellular carcinoma; ns: not significant.

The levels of NTN4 were assessed in patients with HCC based on the Child-Pugh score and depicted in Figure 2. HCC patients with Child-Pugh C score exhibited markedly reduced NTN4 levels compared to Child-Pugh A (p < 0.05) and Child-Pugh B HCC patients (p < 0.05) (Figure 2).

ROC curve analysis of NTN4 in patients with HCC and healthy controls

**Figure 3:**
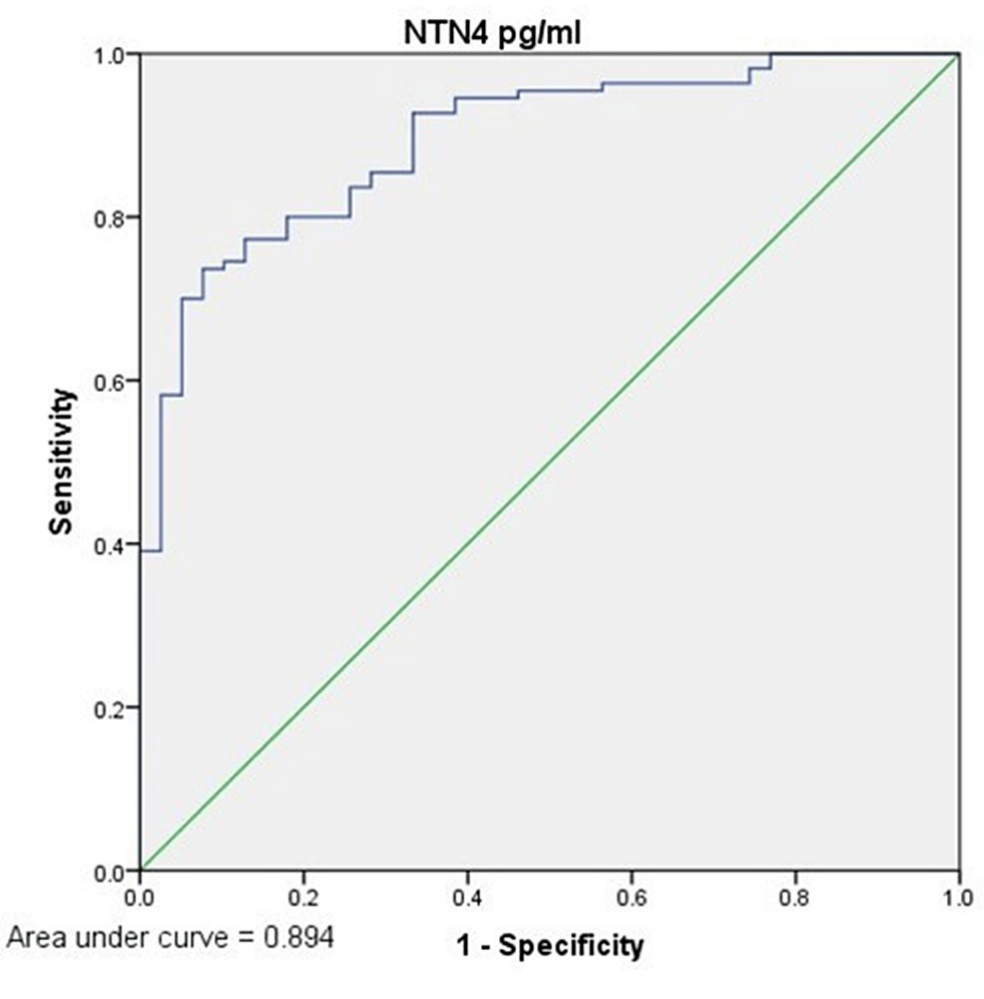
ROC curve analysis of NTN4 in the HCC group and control group ROC curve analysis of NTN4 at a cut-off of 30 pg/ml had a sensitivity of 80% and specificity of 82% with an AUC of 0.894. NTN4: netrin-4; HCC: hepatocellular carcinoma; ROC: receiver operating characteristic; AUC: area under the curve.

ROC curve analysis was performed and AUC was obtained. NTN4 exhibited good discrimination between HCC and healthy controls at the cut-off value of 30 pg/ml, with a sensitivity of 80% and specificity of 82%, and AUC = 0.894 (p = 0.01). The positive predictive value (PPV) for NTN4 was 91%, and the negative predictive value (NPV) was 58% at the cut-off value of 30 pg/ml (Figure 3).

Serum NTN4 levels in patients compared with tumor markers and the stage of the tumor

**Figure 4:**
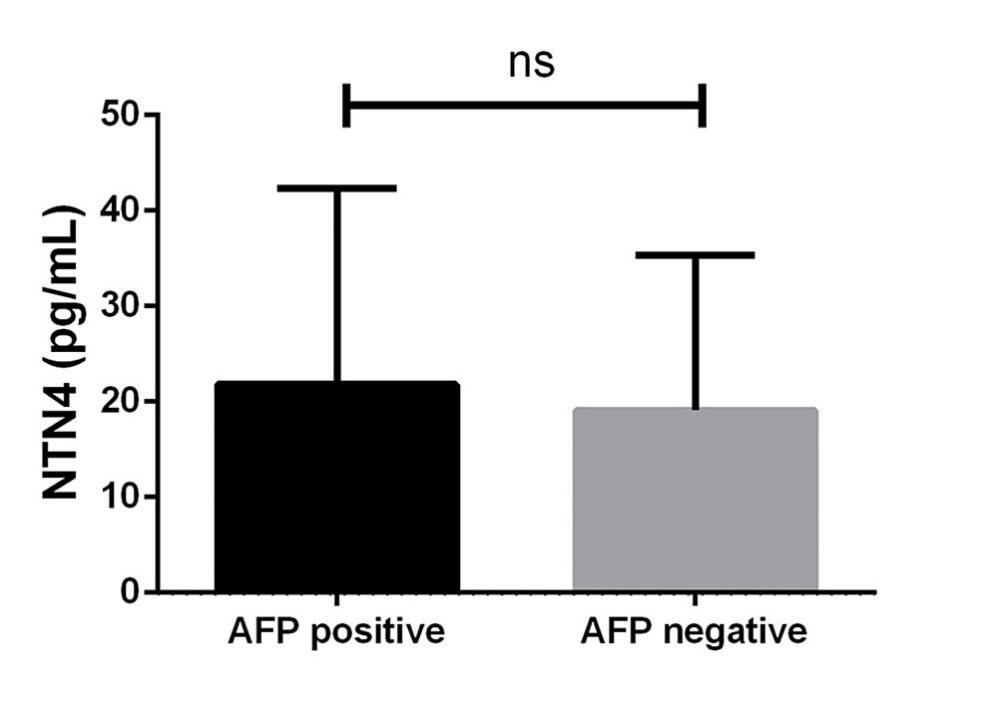
Serum NTN4 levels in patients with HCC with AFP positive and AFP negative Values are expressed as mean ± standard deviation. NTN4: netrin-4; HCC: hepatocellular carcinoma; AFP: alpha-fetoprotein; ns: not significant.

As the AFP level is a known factor associated with the aggressiveness of HCC, a subgroup analysis was performed. NTN4 levels were similar in patients with HCC with normal AFP (negative) compared to those with high levels of AFP (positive) (Figure 4 and Table 4). As metastasis and vascular infiltration are known to be associated with disease progression, another subgroup analysis was done and depicted in Table 4. The presence of metastasis and tumor infiltration to the portal vein was found to be significantly associated with reduced NTN4 levels (Table 4).

**Table 4:** Sub-group analysis of NTN4 levels among clinicopathological characteristics of patients with HCC NTN4: netrin-4; HCC: hepatocellular carcinoma; AFP: alpha-fetoprotein; PVI: portal vein invasion; IQR: interquartile range; p-value < 0.05 is statistically significant; * metastasis (intrahepatic and extrahepatic).

Variables	Category	Mean ± SD	Median (IQR)	P-value
AFP (n = 88)	Positive (n = 55) (>20 ng/mL)	32.7 ± 53.6	21.7 (8.7-29.3)	0.39
	Negative (n = 33) (<20 ng/mL)	3.5 ± 44.6	8.5 (6.0-25.9)	
*Metastasis	Present (n = 13)	15.9 ± 10.9	14 (5.3-22.5)	<0.02
	Absent (n = 97)	30.6 ± 54.4	17.1 (7.2-28.8)	
Main PVI vs. others	Present (n = 3)	13.7 ± 8.51	9.0 (8.7-0)	<0.04
	Absent (n = 94)	31.1 ± 55.2	17.5 (7.1-29.0)	
Main PVI +/- branch vein infiltration vs. others	Present (n = 11)	18.9 ± 8.31	22.0 (9.0-23.6)	<0.05
	Absent (n = 86)	32.1 ± 57.6	15.2 (6.3-29.5)	

Association of NTN4 and biochemical parameters in patients with HCC

The correlation coefficients (r) were calculated between NTN4 and biochemical parameters as depicted in Table 5. A statistically negative correlation was observed between serum NTN4 levels and total bilirubin in HCC patients (Table 5). There was no statistically significant correlation between NTN4 and the other biochemical parameters (Table 5).

**Table 5:** Correlation between NTN4 and biochemical characteristics in patients with HCC r = coefficient of correlation; NTN4: netrin-4; HCC: hepatocellular carcinoma; AST: aspartate transaminase; γGT: gamma-glutamyl transferase; ALP: alkaline phosphatase; * p < 0.05 is a statistically significant correlation.

Variable	r	P
Albumin	0.06	0.5
Total bilirubin	-0.27	0.004*
Total protein	-0.01	0.88
AST	-0.06	0.51
γGT	0.11	0.22
ALP	0.10	0.25

## Discussion

Hypervascularity characterized by angiogenesis and vasculogenesis has been reported to contribute significantly to the pathophysiology of HCC [17]. While many angiogenesis-related markers have been studied in recent years, only a few of them are associated with disease progression in HCC [18]. In the present study, we reported for the first time that the serum levels of NTN4 were markedly reduced among HCC patients. Notably, we observed the reduction in circulatory levels of NTN4 in both AFP-positive and negative HCC patients, indicating its usefulness in the AFP-negative HCC subgroup of patients. Most importantly, we report that the reduction in NTN4 was associated with major pathological events in HCC such as metastasis, main portal vein invasion, and branch vein infiltration. Additionally, we noted that NTN4 levels were negatively associated with bilirubin levels and were markedly reduced in HCC patients with Child-Pugh C score, indicating its association with advanced liver injury and poor prognosis. Our findings indicated that NTN4 is a novel biomarker exhibiting good predictivity for HCC, and is associated with the progression of the disease.

In the present study, the median age of patients with HCC was 60 (50-65) years, which was higher compared to controls 32 (27-36) years. The sex ratio was similar in both groups although there were more males in both groups, which is the expected pattern in HCC and cirrhosis patients. Total bilirubin, AST, ALP, and GGT are some of the most commonly used functional markers in the clinical diagnosis of hepatic dysfunction and injury [19]. Their increased concentrations are found in many acute and chronic liver ailments and are also associated with HCC [20]. A previous study has shown evidence that AST, ALT, and GGT were elevated in 90% of diagnosed patients with HCC, while half of the patients also exhibited an increase in bilirubin or liver-specific ALP concentrations [19], indicating that they can also be treated as additional but highly non-specific markers in HCCs. In the current study, the serum concentration of direct and total bilirubin, AST, ALP, GGT, and total protein was significantly elevated in patients with HCC compared to controls. We also found decreased albumin concentrations in patients with HCC compared to controls.

NTN4, a novel member of the netrin family, is a secreted protein that regulates angiogenesis [13]. Interestingly, NTN4 has been observed to serve as a bi-functional modulator in the angiogenic process, exhibiting both pro- and anti-angiogenic activity that differs according to the type of cancer [12]. NTN4 likely plays an essential role in controlling tumor growth and metastasis. Recent evidence has suggested that NTN4 acts as a tumor suppressor in a range of malignancies affecting the breast, pancreas, prostate, cervical, and colon [21-23]. At a tissue level, NTN4 expression was downregulated in cervical cancer tissues compared to normal controls, implying that NTN4 may play a protective role in cervical cancer pathogenesis [24]. On the contrary, studies have also reported high expression of NTN4 at both the tissue level and circulatory level in gastric cancer patients, where elevation in NTN4 was associated with the severity of pathological stages of gastric cancer [25]. Similarly, few studies have established that NTN4 is significantly upregulated in breast carcinoma effusions compared to patient-matched solid primary and metastatic tumors suggesting that it may be of biological significance along with its prognostic significance [22]. To the best of our knowledge, no previous study has explored the circulating levels of NTN4 in HCC patients. In the present study, we observed decreased levels of serum NTN4 in patients with HCC compared to healthy controls. The decrease in NTN4 levels might be attributed to the concentration-dependent biphasic function of NTN4. At low physiological ligand concentrations, NTN4 has been observed to promote cell survival and migration, whereas, at high concentrations, it behaves as an anti-angiogenic agent to inhibit tumor growth [12]. Findings from experimental models of primary tumor and metastasis in mice indicated that overexpression of NTN4 reduced the recurrence of the tumor as well as metastasis after surgical resection [26]. Further studies are warranted to understand if NTN4 has therapeutic potential against tumor growth and metastasis in HCC.

To further investigate whether NTN4 is involved in the progression of HCC, we explored the association of levels of NTN4 with clinicopathological characteristics of HCC. Unlike many other tumors, angiogenesis is the dominant mechanism of initiation, development, and progression in HCC lesions, a feature that is used for HCC diagnosis and grading. Our study indicated that low NTN4 levels were found to be associated with aggressive HCCs with portal vein invasion, branch vein infiltration, and metastasis. The association of circulating NTN4 levels with the aggressiveness of HCC tumors could be suggestive of the involvement of NTN4 in the development and progression of HCC. Additionally, we identified that HCC patients with Child-Pugh score C had significantly lower NTN4 levels compared to Child-Pugh score A and B patients, indicating the prognostic value of NTN4 in predicting advanced stages of liver injury in HCC patients with Child-Pugh C cirrhosis. In previous studies, it has been reported that the presence of elevated bilirubin levels was associated with portal vein thrombosis, metastasis, and poor prognosis in HCC patients [27]. In the present study, we observed that NTN4 levels were negatively associated with total bilirubin in HCC patients, suggesting that reduced serum NTN4 could be a predictor of poor prognosis in HCC. Furthermore, our ROC analysis demonstrated that at the cut-off value of 30 pg/mL, NTN4 exhibited robust predictive potential for HCC.

Circulating biomarkers have a promising role in the screening and diagnosis of cancer owing to the minimally invasive nature of the test, compared to invasive tissue biopsy. Serum AFP level is the most widely used biomarker in HCC worldwide. However, AFP's reported sensitivity and specificity for early detection and prognosis in HCC patients are inconsistent. AFP has a sensitivity of 60-80% at a lower cut-off value of 20 ng/ml of serum in HCC detection and has an upper specificity level of more than 90% at a higher cut-off value of 200-400 ng/ml [28,29]. AFP levels in serum showed good accuracy in HCC diagnosis, and the threshold of AFP with 400 ng/ml was better than that of 200 ng/ml in terms of sensitivity and specificity, irrespective of its use alone or in combination with ultrasound. Thus, the normal level of AFP for the diagnosis of HCC is seen to vary widely. Furthermore, an increase in serum AFP level (20-200 ng/ml) is identified in a significant percentage of patients with chronic liver disease, including 5-58% of patients with chronic hepatitis, and 11-47% of patients with cirrhosis. Of note, even some histologically proven HCC tumors are regarded as AFP-negative HCCs. Further, the utility of AFP in HCC screening is limited, since it does not allow distinguishing between cancerous lesions, and other benign liver pathologies, especially in early stages, hence resulting in a high proportion of false positives and false negatives. In the current study, we found that 62.5% of HCC patients had elevated (>20 ng/mL) serum AFP levels, while 37.5% had normal (<20 ng/mL) levels. A previous report revealed that AFP was not an optimal marker for the early detection of HCC. Lower diagnostic efficacy of AFP in the advanced stage of HCC has also been reported [28,30]. In our study, NTN4 levels were found to be reduced in both sub-groups of HCC patients with normal and abnormal AFP. Considering that NTN4 was altered even in AFP-negative HCCs, it is indicative of the usefulness of NTN4 as a biomarker in the AFP-negative HCC subgroup, which may also serve as a solution to circumvent the moderate levels of sensitivity and specificity of AFP.

Our study's limitations include that it is a single-center designed study and has a lack of generalizability based on the study setting. There have been few clinical studies on NTN4's effectiveness and mechanism, and there is a lack of preclinical data on NTN4 in human patients with HCC. The pathway of the NTN4 is not clear. Additionally, the absence of randomized controlled trials makes it challenging to establish a cause-effect relationship compared to existing therapies or placebo.

## Conclusions

To the best of our knowledge, this is the first study to report a reduction in circulating NTN4 levels in HCC patients. NTN4 levels can be measured by ELISA, thus making it a minimally invasive biomarker for screening, diagnosis, and prognosis of HCC. Unlike AFP, NTN4 is a marker directly related to angiogenesis, which is the basic and typical tumorigenic mechanism of HCC. The fact that contrast USG, contrast CT, and MRI use the property of vascularity and angiodensity of HCC lesions further underlines the utility of NTN4, which itself is a biomarker of the same features. The findings of this study may further be confirmed at the tissue level, which could shed light on its involvement in various stages of HCC pathogenesis. Additionally, the estimation of tumor number and volume, and its association with NTN4 levels might be useful in further uncovering the prognostic value of NTN4 value in HCC patients.
